# Gene Silencing and Antigenic Variation in Malaria Parasites

**DOI:** 10.1100/tsw.2001.369

**Published:** 2001-11-13

**Authors:** Kirk W. Deitsch

**Affiliations:** Joan and Sanford I. Weill Medical College of Cornell University, New York, NY 10021, USA

**Keywords:** malaria, cytoadherence, transcription, chromatin structure, var

## Abstract

Malaria remains one of the most important infectious diseases in the world today, infecting 300 to 500 million people yearly and resulting in 1 to 2 million deaths, primarily of young African children[1]. The most severe form of this disease is caused by infection with the mosquito borne protozoan parasite Plasmodium falciparum. This parasite lives by invading and multiplying within the red blood cells of its host, causing disease through anemia resulting from red cell destruction, and also through modifications made to the surface of infected red cells. These modifications make infected cells cytoadherent or “sticky”, allowing them to adhere to the walls of blood vessels, leading to obstruction of blood flow and such clinical manifestations as the often fatal syndrome of cerebral malaria[2]. In addition, parasites are capable of undergoing antigenic variation, a process of continually changing the identity of proteins on the surface of infected cells and thus avoiding the immune response mounted by the host[3]. This process promotes a long term, persistent infection that is difficult to clear.

The proteins responsible for the cytoadherent properties of infected red cells are encoded by a mulitcopy gene family called *var*[4,5,6]. While each parasite contains approximately 50 *var* genes within its genome, only a single copy is expressed at any given time[7,8]. Over the course of an infection, expression switches to alternate copies within the family, resulting in antigenic variation and a persistent infection. The molecular mechanisms by which parasites manage to express only a single *var* gene at a time and how switches in expression are coordinated have remained a mystery since the discovery of the *var* gene family in 1995. However, a recent paper appearing in the August 30 issue of *Nature* provides the first description of regulatory elements important in controlling *var* gene expression and implicates alterations in chromatin structure as a key element in maintaining proper patterns of expression[9].

Since the initial description of the *var* gene family, a great deal of progress has been made toward understanding how parasite-infected red blood cells adhere to the blood vessel walls of their human hosts. Experiments have identified the cell surface receptors responsible for these interactions and have begun to dissect which portions of the parasite surface proteins interact with these receptors to mediate the adherence process[10,11]. However, understanding expression switching between individual members of the gene family has been more difficult. Initial reports showed that control of expression is at the level of transcription, with only a single *var* gene being transcribed and all other copies remaining transcriptionally silent[7,8]. Investigations into the structure of *var* genes showed that they share similar sequences in the regulatory regions upstream of the genes[12], and that these sequences do not change when a silent gene becomes activated[13]. In addition, removal of *var* gene promoter from the chromosome and placing it on a plasmid renders it transcriptionally active, indicating that all *var* genes are capable of being transcribed, but that some ill-defined, chromosome-dependent mechanism maintains all but a single gene in a silent state[13]. The nature of this silent state, however, was not understood.

The recent *Nature* paper provides the first description of the mechanism responsible for silencing all but a single copy of the *var* family. All *var* genes have a similar structure that includes two exons separated by a conserved 800- to 1000-bp intron. Cooperative regulatory elements were discovered within this intron and in the region upstream of the gene. While neither element functions as a silencer separately, when located together they silence expression of the associated *var* promoter. In addition, when placed on a transfected plasmid, these two elements can cooperate to assemble a transcriptionally silent state outside of the environment of the chromosome. The plasmids only become silenced once the transfected cell has gone through S-phase of the cell cycle. This experimental system should allow detailed dissection of the silencing process using easily modified and manipulated plasmids. The characteristics of this silencing phenomenon very closely resemble those described in other organisms where transcriptional regulation depends on modifications in chromatin structure. Chromatin modification is now thought to play a pivotal role in *var* gene regulation and antigenic variation of malaria parasites.

While this work provides a framework for understanding how the majority of the *var* gene family is maintained transcriptionally silent, it does not provide a description for how individual genes are activated. In addition, how parasites coordinate switches in expression remains a mystery. Therefore, considerably more work is required before a complete understanding of the mechanisms regulating antigenic variation in these parasites is obtained. However, with the discovery of DNA elements involved in this process as well as an appreciation for the role chromatin structure plays in controlling *var* gene transcription, gains in understanding are likely to be rapidly achieved. The study of gene regulation by chromatin modification is an extremely dynamic subject, with new discoveries reaching the literature at a rapid pace on such topics as developmental gene expression patterns, epigenetic inheritance, and cellular memory. Advances in understanding this process in malaria parasites will therefore contribute to this rapidly advancing field.
